# A Computational Phenotype of Disrupted Moral Inference in Borderline Personality Disorder

**DOI:** 10.1016/j.bpsc.2020.07.013

**Published:** 2020-12

**Authors:** Jenifer Z. Siegel, Owen Curwell-Parry, Steve Pearce, Kate E.A. Saunders, Molly J. Crockett

**Affiliations:** a Department of Psychology, Columbia University, New York, New York; b Department of Psychiatry, University of Oxford, Warneford Hospital, Oxford, United Kingdom; c Oxford Health National Health Service Foundation Trust, Warneford Hospital, Oxford, United Kingdom; d Department of Psychology, Yale University, New Haven, Connecticut

**Keywords:** Bayesian inference, Belief updating, Borderline personality disorder, Computational psychiatry, Moral impression formation, Social behavior

## Abstract

**Background:**

Borderline personality disorder (BPD) is a serious mental disorder characterized by marked interpersonal disturbances, including difficulties trusting others and volatile impressions of others’ moral character, often resulting in premature relationship termination. We tested a hypothesis that moral character inference is disrupted in BPD and sensitive to democratic therapeutic community (DTC) treatment.

**Methods:**

Participants with BPD (*n* = 43; 20 untreated and 23 DTC-treated) and control participants without BPD (*n* = 106) completed a moral inference task where they predicted the decisions of 2 agents with distinct moral preferences: the “bad” agent was more willing than the “good” agent to harm others for money. Periodically, participants rated their subjective impressions of the agent’s moral character and the certainty of those impressions. We fit a hierarchical Bayesian learning model to participants’ trialwise predictions to describe how beliefs about the morality of the agents were updated by new information.

**Results:**

The computational mechanisms of moral inference differed for patients with untreated BPD relative to matched control participants and patients with DTC-treated BPD. In patients with BPD, beliefs about harmful agents were more certain and less amenable to updating relative to both control participants and participants who were treated with DTC.

**Conclusions:**

The findings suggest that DTC may help the maintenance of social relationships in BPD by increasing patients’ openness to learning about adverse interaction partners. The results provide mechanistic insights into social deficits in BPD and demonstrate the potential for combining objective behavioral paradigms with computational modeling as a tool for assessing BPD pathology and treatment outcomes.

SEE COMMENTARY ON PAGE 1075

Borderline personality disorder (BPD) is a serious mental disorder affecting up to 5.9% of the general population (1). Marked disturbances in interpersonal relationships constitute one of the core symptom domains of BPD, including difficulties with trust and forgiveness often resulting in premature relationship termination (2, 3, 4). Difficulties related to interpersonal relationships contribute to substantial economic and societal costs including high rates of suicide and intensive use of high-cost medical care (5, 6, 7, 8). Longitudinal studies indicate that symptoms related to interpersonal relationships are among the hardest to treat; serious social deficits often persist even after years of rigorous and resource-exhaustive treatment (9, 10, 11, 12). Research identifying the mechanisms of impaired social functioning in BPD is therefore paramount for relieving interpersonal and societal burdens.

Several possible explanations have been proposed for why patients with BPD exhibit a poor ability to maintain interpersonal relationships. For instance, building and maintaining successful social relationships depends on the ability to build accurate representations of others’ mental states (e.g., intentions, beliefs, desires); however, research suggests that patients with BPD may be limited in their ability to accurately perceive social signals and model the intentions of others (2). Notably, adaptive social functioning also depends on the ability to continuously update representations of others through social learning (13). A growing body of theoretical and empirical work suggests that impaired social learning plays an important role in interpersonal disturbances in BPD, including difficulties trusting others (3,14,15). Here, we consider an aspect of social learning that is especially relevant to forming and maintaining relationships: inferring others’ moral character (16,17); that is, whether they are helpful and trustworthy or harmful and untrustworthy.

We introduce a novel computational assay of moral inference to investigate how patients with BPD form beliefs about the moral character of others and incorporate new information into existing beliefs. Previous research using these methods indicates that healthy adults hold more uncertain and less rigid beliefs when inferring a “bad” moral character relative to a “good” moral character (17,18). This work implemented a Bayesian inference framework where beliefs are updated in proportion to their uncertainty (19), such that more uncertain beliefs are updated more rapidly. Consequently, more uncertain negative beliefs about others’ morality enables those beliefs to be rapidly updated from new information, which is hypothesized to reflect an adaptive mechanism for sustaining relationships when others sometimes behave badly. Thus, holding negative moral beliefs with some degree of uncertainty may be an important aspect of healthy social functioning. Given that individuals with BPD often hold grudges and have difficulty forgiving others (4,20), we tested a hypothesis that relative to control participants without BPD, patients with BPD have more certain and rigid beliefs about harmful agents and therefore lack this adaptive mechanism for forgiveness that may help sustain relationships.

Understanding the mechanisms underlying interpersonal problems in BPD is essential for developing and assessing effective treatments. Democratic therapeutic community (DTC) treatment is among the most widespread psychosocial treatments for BPD in the United Kingdom; it has a strong focus on developing cooperative strategies to help patients effectively navigate their social environments (21) and has been associated with improvements in social functioning at least 24 months following treatment (22), including more pleasant social relations (23). While DTC aims to help patients learn new strategies for adaptive social functioning, it is unknown how the effects of treatment manifest at the cognitive level. Understanding the cognitive channels through which DTC operates may ultimately help identify which patients may benefit the most from such treatment. To shed light on this question, the present research therefore assessed moral inference in a group of participants with DTC-treated BPD compared to a group of untreated participants with BPD.

## Methods and Materials

### Participants

#### Non-BPD Group

The online crowdsourcing platform Prolific (www.prolific.ac) enabled us to collect a sample of adult participants precisely matched to our patient population who would not qualify for a diagnosis of BPD. This method has the potential to improve the validity and generalizability of research by enabling efficient and low-cost recruitment of comparison groups for unique samples who may come from specific environments (24). Previous research has established that a diverse set of cognitive tasks (such as the Stroop, Flanker, and category learning) show similar results in the lab and online (25). Subjects recruited through online platforms are at least as attentive (26) and consistent (27) in their task performance as participants recruited through college subject pools. Furthermore, a recent study showed that participants recruited through the Prolific platform produced data quality that was higher than comparable online crowdsourcing platforms as well as a university subject pool (28). We aimed to recruit 5 healthy adults who matched each patient with BPD in gender, age (±4 years), and education. We ensured that matched participants received the same variant of the moral inference task as their patient counterpart (i.e., same sequence of trials).

Control participants provided written informed consent after receiving a complete description of the study and were compensated for their time. The Yale University Human Investigation Committee approved the procedures (#2000022385). Participants completed the study on the web application framework Heroku (Salesforce, San Francisco, CA) and were subsequently directed to a Qualtrics survey (Qualtrics, Provo, UT) to complete additional questionnaires to assess clinically relevant personality traits. Previous work has demonstrated that the moral inference task yields comparable results in lab and online settings (17). Control participants completed the McLean Screening Inventory for BPD (see Supplemental Methods) and were excluded from the analysis if they showed clinically relevant BPD symptoms (McLean Screening Inventory score > 6). The final sample of control participants included 106 adults who scored lower than 7 on the McLean Screening Inventory.

#### BPD Group

Participants were treatment-seeking individuals with a primary diagnosis of BPD recruited from an outpatient population. The Structured Clinical Interview for Axis II Disorders (see Supplemental Methods) was administered by trained clinicians to establish BPD diagnosis. Inclusion criteria were diagnosis of BPD, age between 18 and 65 years, not currently being treated in group therapy, no current drug or alcohol dependence, and no psychiatric hospital admission in the preceding month. Individuals were excluded if they had a previous or current neurological condition, were unable to provide informed consent, were pregnant or breastfeeding, or met criteria for an Axis I illness (e.g., anxiety, mood, eating disorders). Nine participants were taking antidepressant or antipsychotic medication or both at the time of participation. The final sample included 20 participants with BPD.

#### DTC Group

Participants with a primary diagnosis of BPD who completed DTC treatment (22) within 3 years prior to recruitment were recruited from the Oxfordshire and Buckinghamshire Complex Needs Service database. As part of the program, participants who found DTC unhelpful or are deemed to not be progressing their therapy would leave the program by mutual consent. Eligible participants were contacted by postal mail and sent a copy of the information sheet along with an invitation to participate in the study. The Structured Clinical Interview for Axis II Disorders was administered to interested individuals by trained clinicians to establish BPD diagnosis. Inclusion criteria were diagnosis of BPD, age between 18 and 65 years, completed DTC at the Oxfordshire and Buckinghamshire Complex Needs Service (22) within the past 3 years, and no current drug or alcohol dependence. Individuals were excluded on the same basis as participants in the untreated BPD group. Eleven participants were taking antidepressant or antipsychotic medication or both at the time of participation. The final sample included 23 participants with BPD who had completed DTC treatment.

Behavioral testing of participants with BPD (untreated BPD and DTC-treated groups) took place at the University of Oxford, Department of Psychiatry. We used the Borderline Evaluation of Severity Over Time scale to assess the severity of BPD symptomology in participants with BPD at the time of participation (Supplemental Methods). Participants provided written informed consent after receiving a complete description of the study and were compensated for their time. The study was approved (14/SC/1430) by the local National Health Service ethics committee in Oxford.

### Moral Inference Task

**Figure 1:**
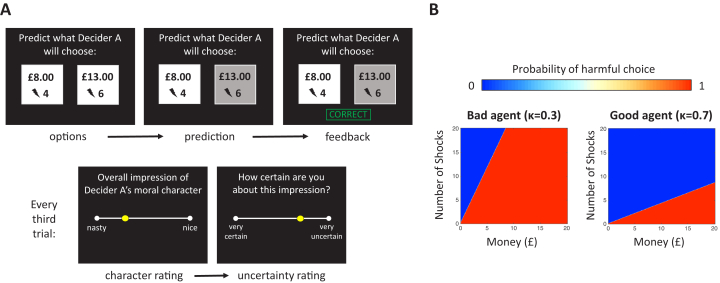
Moral inference task. **(A)** Schematic representation of the moral inference task. Participants predicted sequences of choices for 2 agents (Decider A and Decider B). On each trial the agent chose between 2 options: more shocks inflicted on another person in exchange for more money or fewer shocks in exchange for less money. After making each prediction, participants observed the agent’s actual choice and received feedback indicating whether their prediction was correct or incorrect. Every third trial participants rated their subjective impression about the agent’s moral character (ranging from nasty to nice) and how certain they were about their impression. **(B)** Heat maps summarize the good and bad agents’ probabilities of choosing the more profitable and harmful option as a function of the amount of money gained and number of shocks inflicted.

In the moral inference task (17), participants predicted and observed the choices of 2 agents (called “Decider A” and “Decider B”) who repeatedly decided whether to inflict painful electric shocks on a victim in exchange for various amounts of money (Figure 1A). The 2 agents differed substantially in their moral preferences: the “good” agent required more compensation to inflict pain on others than the “bad” agent (Figure 1B). Periodically, participants rated their subjective impressions of the agent’s morality (from 0 = “nasty” to 100 = “nice”), and the certainty of those impressions (from 0 = “very uncertain” to 1 = “very certain”). Before observing any of the agent’s choices, participants additionally indicated how nasty or nice they expected the agent would be and how certain they were. This provided an indication of participants’ prior expectations about people’s moral character in general and their confidence in those prior expectations. We confirmed that the groups were equally motivated to learn about the agents and predict their decisions (see Supplemental Results).

### Computational Modeling

We fit a generative Bayesian reinforcement learning model (17, 18, 19,29) to participants’ trial-by-trial predictions. The model identified participant-specific parameters to describe how the participants updated their beliefs about the morality of the agents, as described by Siegel *et al.* (17). In the model, beliefs about an agent’s moral preference (i.e., the exchange rate between money and shocks) are updated from new information with dynamic learning rates. Learning rates capture the weight participants place on new information over prior beliefs when updating beliefs on the current trial. When prior beliefs are less precise, learning rates are higher, such that less precise beliefs are more heavily updated from new information. Random-effects Bayesian model selection indicated that our model with a dynamic learning rate was preferred over 1) a model where beliefs were updated by new information with a fixed learning rate, and 2) a model where beliefs were updated by new information with separate fixed learning rates for positive (helpful) and negative (harmful) information (see Supplemental Results). Additionally, the proportion of participants whose data was best explained by our model with a dynamic learning rate did not significantly differ across BPD, control, and DTC groups (χ^2^_2,149_ = 3.044, *p* = .218) (see Supplemental Results).

### Analysis

We used robust linear regression models with bisquare weighting functions to analyze standardized learning rates, subjective character impression ratings, and certainty ratings (using the RobustOpts setting in the fitlm function in MATLAB; The MathWorks, Inc., Natick, MA). Certainty ratings were reverse scored such that higher values indicated greater uncertainty in subjective impressions of the agents’ moral character. Because learning rates and subjective ratings evolve over time, we initially considered whether groups differed as a function of time dynamics (i.e., trial number) and found no evidence to support this prediction. Consequently, regression models included the effects of agent (bad, good), group (BPD, non-BPD, DTC), and their interaction, controlling for trial number. Further analyses used two-sided nonparametric statistical tests that do not make any assumptions about the underlying distributions of variables (e.g., Wilcoxon rank-sum test).

## Results

An omnibus test for group × agent interactions, where group was coded as a dummy variable (with untreated BPD as the reference group), found significant differences in the effect of agent between groups on uncertainty ratings (non-BPD, β = .264 ± .080, *t* = 3.310, *p* < .001; DTC, β = .266 ± .100, *t* = 2.665, *p* = .008) and learning rates (non-BPD, β = .113 ± .025, *t* = 4.607, *p* < .001; DTC, β = .319 ± .031, *t* = 10.355, *p* < .001) (see Supplemental Results for full analyses). For clarity, here we first present comparisons between participants with untreated BPD and control participants, followed by comparisons between untreated BPD and DTC-treated groups.

### Moral Inference in BPD

We analyzed data in the moral inference task for participants with untreated BPD and control participants who were matched for gender, age, education, and self-report psychopathy, but significantly differed in levels of clinically relevant personality traits (Table 1).

**Table 1:** Participant Demographic Information, BPD vs. Non-BPD

	Untreated BPD (*n* = 20)		Non-BPD (*n* = 106)		*z* Statistic	*p* Value
	Mean	SEM	Mean	SEM		
Age on Date of Participation, Years	39.500	2.561	40.957	1.140	−0.612	.540
Highest Level of Education, No. of Degrees	2.412	0.195	2.587	0.094	−0.861	.389
Psychopathy Score	42.053	2.024	38.387	0.795	1.437	.151
Personality Inventory for DSM-V Score	39.950	3.042	18.740	1.202	5.269	<.001

BPD, borderline personality disorder.

**Figure 2:**
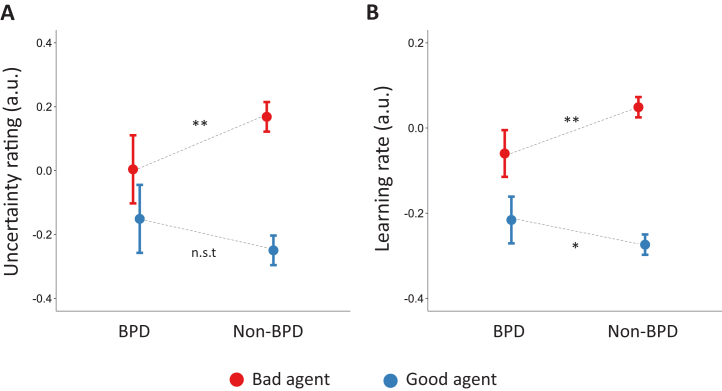
Negative beliefs are more certain and slower to update in untreated participants with borderline personality disorder (BPD) relative to non-BPD control participants. **(A)** Relative to control participants, participants with BPD held less uncertain impressions of the bad agent. **(B)** Participants with BPD were slower to update beliefs about the bad agent following new information. Error bars represent 95% confidence intervals. ∗*p* < .05; ∗∗*p* < .01; nonsignificant trend (n.s.t., *p* < .1), where significance refers to the interaction between group and agent in our regression models. a.u., arbitrary units.

We first inspected participants’ subjective impressions of the agents’ moral character, and their uncertainty about those impressions. While there were no differences between participants with BPD and control participants in average character impressions (see Supplemental Results), group differences emerged for the uncertainty ratings. Consistent with prior findings (17), participants overall held more uncertain impressions of the bad agent than of the good agent (main effect of agent, β = .418 ± .032, *t* = 13.099, *p* < .001); however, this effect was substantially reduced in participants with BPD (interaction between agent and group, β = −.263 ± .080, *t* = −3.284, *p* = .001) (Figure 2A). Relative to control participants, participants with BPD held less uncertain impressions of the bad agent (β = −.162 ± .058, *t* = −2.805, *p* = .005), but were similarly uncertain about their impressions of the good agent (β = .098 ± .055, *t* = 1.761, *p* = .078).

Learning rate data were consistent with the uncertainty rating data. Overall, participants updated beliefs faster for the bad agent than for the good agent (main effect of agent, β = .323 ± .017, *t* = −18.601, *p* < .001); however, this effect was substantially smaller in participants with BPD (interaction between agent and BPD group, β = −.167 ± .044, *t* = −3.827, *p* < .001) (Figure 2B). Specifically, participants with BPD were slower to update beliefs about the bad agent (β = −.109 ± .034, *t* = −3.222, *p* = .001) and faster to update beliefs about the good agent (β = .062 ± .027, *t* = 2.287, *p* = .022) relative to control participants. The findings suggest that BPD is associated with more confident and less flexible beliefs about harmful agents, but less confident and more flexible beliefs about helpful agents. A supplementary analysis (using data across all BPD groups) revealed that BPD symptom severity moderated the observed effects, such that participants with more severe BPD symptoms expressed less uncertain impressions of the bad agent and more uncertain impressions of the good agent (see Supplemental Results).

Participants with BPD indicated more pessimistic expectations before observing any of the agents’ choices than control participants (*z* = −2.491, *p* = .013), though participants with BPD and control participants were similarly certain about their expectations (*z* = −0.327, *p* = .743). Thus, a plausible explanation for the observed pattern of results is that the good agent violated the expectations of participants with BPD to a greater degree than the bad agent. Given our particular model, this could make beliefs about the good agent more amenable to Bayesian updating in BPD, by which belief updates are optimized to minimize surprise (19). Previous research indicates that healthy adults are able to override externally generated prior expectations and rapidly adjust their learning as a function of moral character information (17), prioritizing belief updating for putatively bad agents. We replicated this finding in the control participants (see Supplemental Results). However, analyses suggested that unlike healthy adults, learning may be especially sensitive to prior expectations in BPD (see Supplemental Results).

### Moral Inference in Participants With DTC-Treated BPD

Next, we compared performance on the moral inference task for participants with DTC-treated and untreated BPD who were matched for gender, age, education, self-report psychopathy, and clinically relevant personality traits (Table 2). We confirmed that the severity of BPD symptomology in DTC-treated participants was significantly lower than in participants with untreated BPD (borderline evaluation of severity over time, *z* = 3.690, *p* < .001).

**Table 2:** Participant Demographic Information, Untreated vs. DTC-Treated BPD

	Untreated BPD (*n* = 20)		DTC-Treated (*n* = 23)		*z* Statistic	*p* Value
	Mean	SEM	Mean	SEM		
Age on Date of Participation, Years	39.500	2.561	41.609	2.205	−0.573	.567
Highest Level of Education	2.412	0.195	2.632	0.211	−0.748	.455
Psychopathy	42.053	2.024	40.217	2.628	0.999	.318
Personality Inventory for DSM-V	39.950	3.042	33.478	3.029	1.572	.116
Borderline Evaluation of Severity Over Time	41.444	1.975	26.867	1.956	3.690	<.001

BPD, borderline personality disorder; DTC, democratic therapeutic community.

DTC-treated participants expressed more favorable impressions in general than the untreated participants (main effect of group, β = .146 ± .046, *t* = 3.197, *p* = .001). This group difference appeared to be primarily driven by impressions of the good agent (interaction between agent and group, β = −.236 ± .064, *t* = −3.668, *p* < .001), such that the DTC-treated participants, relative to untreated participants, expressed more favorable impressions of the good agent (β = .151 ± .043, *t* = 3.507, *p* < .001). Group differences in impressions of the bad agent did not reach significance (β = −.090 ± .048, *t* = −1.869, *p* = .062).

**Figure 3:**
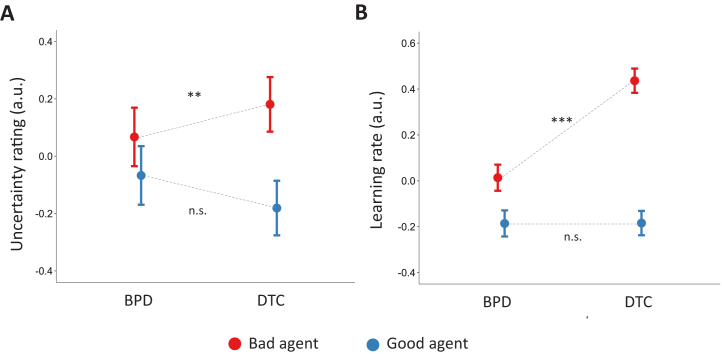
Negative beliefs are more uncertain and faster to update in democratic therapeutic community (DTC)-treated participants than untreated participants with borderline personality disorder (BPD). **(A)** Relative to untreated BPD, DTC treatment was associated with more uncertain impressions of the bad agent. **(B)** DTC-treated participants were faster to update beliefs about the bad agent from new information than untreated participants with BPD. Error bars represent 95% confidence intervals. ∗∗*p* < .01; ∗∗∗*p* < .001; not significant (n.s., *p* > .1), where significance refers to the interaction between group and agent in our regression model. a.u., arbitrary units.

Turning to the uncertainty of impressions and learning rates, we found that DTC-treated participants, relative to untreated participants, showed more uncertain impressions of the bad agent (β = .188 ± .067, *t* = 2.802, *p* = .005) (Figure 3A) and faster learning rates for the bad agent (β = .543 ± .040, *t* = 13.698, *p* < .001) (Figure 3B), as indicated by significant interactions between agent and group for both measures (uncertainty ratings, β = .277 ± .095, *t* = 2.904, *p* = .003; learning rates, β = .589 ± .052, *t* = 11.588, *p* < .001) (see Supplemental Results for full regression analyses). No group differences were observed on impression uncertainty or learning rates for the good agent (uncertainty, β = −.081 ± .068, *t* = −1.196, *p* = .232; learning rates, β = −.030 ± .030, *t* = −0.989, *p* = .323). Thus, DTC treatment was associated with increased uncertainty and more flexible beliefs about the bad agent, specifically.

DTC-treated and untreated participants had similar expectations about the agents’ morality (*z* = 0.585, *p* = .559) and were similarly certain about their expectations (*z* = 0.585, *p* = .559). Negative expectations therefore do not account for the observed group differences in moral inference. For completeness, we investigated whether prior expectations covaried with the interaction between group and agent and report the results in the Supplement. Overall, we found that even though DTC-treated and untreated participants had similar moral expectations, the groups differed in how expectations subsequently shaped learning.

In the present study, many participants were taking psychotropic medication at the time of participation. It is possible that group differences in pharmacological treatments, rather than DTC treatment, drove increased flexibility and belief updating for the bad agent. However, we observed a similar interaction between agent and group on uncertainty ratings and learning rates when controlling for medication use (uncertainty, β = .277 ± .095, *t* = 2.898, *p* = .004; learning rates, β = .577 ± .050, *t* = 11.441, *p* < .001) (see Supplemental Results for full regression analyses).

## Discussion

Here we identify a computational phenotype that may characterize some aspects of BPD pathology and is sensitive to a common treatment. Unlike healthy adults, who maintain flexibility in their beliefs about potentially harmful social partners, participants with BPD hold more certain negative beliefs about others and are slower to update those beliefs. DTC treatment was associated with more uncertain, flexible beliefs about putatively harmful social partners, suggesting that DTC may improve social interactions in BPD by increasing participants’ openness to learning about partners who exhibited potentially threatening social interactions.

Cumulatively, our results could provide a computational framework for understanding seemingly paradoxical findings of both volatility and rigidity of social beliefs in BPD. Our observation of more rigid negative beliefs in BPD is consistent with past reports that patients with BPD show slower learning rates in a task that requires learning about the probability of social and nonsocial cues, less conciliatory social behavior following a rupture of trust (2), and difficulty forgiving others (4). We also found some evidence that participants with BPD hold less certain positive beliefs about others and are faster to update those beliefs. This finding is consistent with the ease patients have in terminating relationships as well as clinical observations that the patient can shift rapidly from a period of admiration to dislike in response to even minor slights (30).

In contrast to past work, by modeling social learning within a Bayesian framework, we are able to consider another important aspect of healthy social cognition. In optimal Bayesian inference, learning is intrinsically tied to prior expectations. Observations that are consistent with prior expectations help reinforce them, while those that are inconsistent may be used to update expectations. However, moral inference departs from Bayesian optimality in an important way: healthy adults maintain more uncertain beliefs about the moral character of putatively bad agents even when observations are consistent with prior expectations (17). We hypothesize that humans have evolved to rapidly discount prior expectations to adapt learning according to moral information. This feature of healthy social cognition provides the flexibility to promptly update beliefs about bad agents when those beliefs turn out to be wrong, preserving social relationships in the wake of accidental harms.

One possibility is that BPD impacts cognitive processes important for the ability to adapt learning as a function of moral information. In turn, patients may rely heavily on pessimistic prior expectations born from adversity and volatility in their social environment (31, 32, 33). While the ability to rapidly discount externally generated prior expectations in moral inference may be advantageous in environments where social partners are consistently trustworthy, it can be costly when partners behave unpredictably. By shutting down the gateway for learning when behavior misaligns with antisocial expectations, rigidity then provides a protective mechanism that prevents responding to unreliable social cues. We found evidence consistent with the hypothesis that participants with untreated BPD may be especially reliant on pessimistic expectations in moral inference (outlined in Supplemental Results). However, more work is needed to assess whether abnormal moral inference in BPD can be explained by an increased tendency to rely on pessimistic prior expectations.

DTC offers a safe environment for patients with BPD to learn the skills necessary for successful social functioning and has shown promise in ameliorating social difficulties (22). Our findings suggest that DTC may positively impact social interactions by increasing patients’ openness to learning about potentially threatening social interaction partners, allowing information to be integrated over longer time scales before establishing a negative evaluation. On the other hand, whether DTC impacts learning about positive social interaction partners, and the development of stable positive beliefs, remains uncertain. If mentalization-based therapies have an impact on epistemic trust, as recent models are proposing (14,34), it may be especially effective in addressing difficulties in establishing stable positive social beliefs in BPD. By applying and comparing this measure in alternative treatment groups, we can better understand the mechanisms through which they impact moral inference and social functioning. Additionally, the research methods presented here can help future studies determine whether the impact of DTC on moral inference can be attributed to the specific therapeutic environment or is a more general result of recovery from BPD symptoms that may arise from any treatment modality.

A major limitation of this study is that we chose to investigate moral inference in individuals with a primary diagnosis of BPD, rather than considering symptom clusters associated with a primary diagnosis of BPD. However, it is likely that these disruptions to moral inference are not specific to BPD as a category, but rather relate to aspects of cognition that are predictive of a variety of disorders. This initial study provides a proof of concept that we have identified a dimension of cognition that distinguished between patients with BPD and a sample of healthy control participants. Future work should apply this measure to larger and more diverse samples to characterize how moral inference relates to a variety of other cognitive and affective dimensions that are relevant for psychiatric symptoms. Additionally, data collection in the present study relied on the availability of a small population of participants with BPD who had completed DTC treatment, and a matched set of treatment-seeking participants with BPD. Given that our sample size was determined by participant availability, further studies are needed to replicate the present findings and assess their generalizability to the larger population of individuals diagnosed with BPD.

A final limitation is that a number of DTC-treated and untreated patients were receiving psychotropic medication. Preliminary analyses (outlined in Supplemental Results) suggest that our main findings remain significant after accounting for medication use. Nonetheless, future work should investigate moral inference in a sample of patients with BPD who are free from psychotropic medication and evaluate whether, in a larger sample, psychotropic medications influence the BPD computational phenotype that we describe.

Our moral inference paradigm captures some of the richness of BPD pathology and may have significant utility. As is the case for all disorders, clinical diagnosis of BPD relies largely on informal observation and subjective self-report. The categorical diagnostic system that relies on these data yields heterogeneous groupings that correspond poorly to disease mechanisms (35). This problem is especially serious for personality disorder, with most patients meeting criteria for multiple diagnoses (36, 37, 38). Indeed, the most common diagnosis for personality disorder patients is “not otherwise specified,” which is provided when a clinician decides a personality disorder is in fact present but the patient is not well described by existing diagnostic categories (37). This highlights the pressing need for better diagnostic tools. The paradigm described here, which can be delivered online and at scale, has the potential to identify the mechanisms by which current treatments act and thus improve them. For instance, the specificity of DTC on learning about adverse social interaction partners raises the possibility that different treatments may improve different aspects of social beliefs in BPD. Using the tools presented here, we may be better equipped to identify individual differences in aberrant moral inference and match patients with treatments best suited for them. Computational modeling of moral inference dynamics may therefore prove a useful tool for investigating longitudinally how aspects of learning and impression updating might predict the course of treatment.

Translating advances in theoretical models of BPD into quantifiable benefits for patients is both conceptually and operationally challenging given the richness of BPD pathology. Tackling this problem requires precise techniques to objectively measure latent cognitive mechanisms that generate observed behavior. Here, we combine a generative model for inferring the morality of others with a moral inference task to provide mechanistic insights into social deficits in BPD. We show that BPD is associated with a specific computational phenotype of moral inference, characterized by rigid negative beliefs about others’ morality. This may impact patients’ ability to forgive others for their misdeeds and impact the maintenance of healthy relationships. DTC may shape social interactions in BPD by decreasing the rigidity of negative beliefs, subsequently increasing patients’ openness to learning about potentially adverse others. Together, the findings demonstrate the potential for combining objective behavioral paradigms with computational modeling as a tool for assessing BPD pathology and treatment outcomes.
