# Supplementary material for: A Computational Phenotype of Disrupted Moral Inference in Borderline Personality Disorder
Source: Biol Psychiatry Cogn Neurosci Neuroimaging. 2020 Dec;5(12):1134–41. doi: 10.1016/j.bpsc.2020.07.013 (PMC7718209; doi:10.1016/j.bpsc.2020.07.013)
Supplement: Supplementary Material [file mmc1.pdf]

A Computational Phenotype of Disrupted Moral Inference in Borderline Personality Disorder

Supplemental Information

SUPPLEMENTAL METHODS

Moral Inference Task

In the Moral Learning Task, participants predicted a series of 50 decisions, made by each of two agents. For each decision, agents chose whether to increase their own profit at the expense of a greater amount of harm, in the form of electric shocks, to an anonymous stranger (**Figure 1a**). Thus, each choice involved choosing between a more harmful option (more money and more shocks) and a less harmful option (less money and less shocks). We simulated the agents to have significantly different preferences towards harming the stranger: one agent was more harmful, accepting less money to increase shocks to the victim ('bad' agent; $0.43 per shock), and the other was less harmful and required more money to increase shocks ('good' agent; $2.40 per shock; **Figure 1b**). After predicting each choice, participants received feedback about their accuracy. Participants did not receive any information about the agents' harm preference prior to the task. Thus, to optimally predict the agents' decisions participants must gather information across trials and learn about the agents' harm preference (i.e., the agent's exchange rate between money and shocks). For complete details about the task and how the agent's choices were simulated, see Siegel et al. 2018 (1).

On every third trial participants indicated their general impression of the agent's moral character (from 0 = *nasty* to 100 = *nice*) and how *certain* they were about their impression (from 0 = *very uncertain* to 100 = *very certain*). This provided us, for each subject and agent, a trajectory of trial-wise *subjective impression ratings* and *uncertainty ratings*. Before observing any of the agent's choices, participants additionally indicated how nasty or nice they *expected* the agents would be and how certain they were. This provided an indication of participants' prior expectations about people's moral character in general and their confidence in those prior expectations.

Hierarchical Gaussian Filter (HGF)

The HGF (2,3) draws on the idea that the brain has evolved to process information in a manner that approximates statistical optimality given individually varying priors about the nature of the process being predicted; effectively maintaining and updating a generative model of its inputs to infer on hierarchically organized hidden states. A basic feature of the model is the division into perceptual and response models, which describes both how participants update their beliefs about hidden states from inputs (perceptual model) and how they are used to make predictions (response model).

Perceptual model. Our model comprises only two hidden states x_1^i and x_2^i, where i signifies the trial index. The first state, x_1, is time-varying and denotes the agent's upcoming choice. x_1 is binary because there are only two options that the agent can choose: the more harmful option (greater profit for the self and more shocks for the victim) or the less harmful option (less profit for the self and fewer shocks for the victim). The probability that an agent will choose the more harmful option ($x_1^i = 1$) versus the less harmful option ($x_1^i = 0$) is governed by the next state in the hierarchy, x_2. x_2 is a continuous state evolving over time as a Gaussian random walk, and signifies the belief about the agent's exchange rate between money and pain. The hierarchical coupling between x_1^i and x_2^i explains that a participant's prediction about an agent's choice on trial *i* is dependent on their current belief about that agent's exchange rate between money and pain, defined as a probability density.

The conditional probability of x_1 given x_2 is described in **Equation 1**.

Equation 1

$$p(x_1|x_2) = s(x_2)^{x_1}\big(1 - s(x_2)\big)^{1-x_1} = \text{Bernoulli}(x_1; s(x_2))$$

Where $s(\cdot)$ is a logistic sigmoid (softmax) function:

Equation 2

$$s(x) \stackrel{\text{def}}{=} \frac{1}{1 + \exp(-x)}$$

The temporal evolution of x_2 is governed by a participant-specific parameter ω, which allows for inter-individual differences in belief updating. Thus, ω captures inter-individual variability in the rate at which beliefs evolve over time, and consequently how rapidly people update their beliefs about the agent's harm aversion across all trials. As ω approaches ∞ beliefs become increasingly unstable and new information is favored over historical information. Conversely, as ω approaches -∞ beliefs become increasingly stable, so greater weight is instead placed on historical information. Given ω and the previous value (with time index $i - 1$) of x_2, we now have the generative model for the current values (with time index i) of x_1 and x_2 in **Equation 3** (for details see (2)).

Equation 3

$$p\big(x_1^i, x_2^i, |\omega, x_2^{i-1}\big) = p\big(x_1^i \big| x_2^i\big) p\big(x_2^i \big| x_2^{i-1}, \omega\big)$$

with

Equation 4

$$p\big(x_2^i \big| x_2^{i-1}, \omega\big) = \mathcal{N}\big(x_2^i; x_2^{i-1}, \exp(\omega)\big)$$

Model inversion was used to optimize the posterior densities over hidden states, x_1 and x_2, and parameter ω. Participants' posterior beliefs were represented by probability distributions with mean μ and variance σ. Variational Bayesian inversion yields a simple update equation under a mean-field approximation, where beliefs are updated as a function of precision-weighted prediction errors. For the present study we focus on the update at level 2 of the hierarchy (2).

Equation 5

$$\Delta\mu \propto \sigma_2 \delta_1^i$$

with

Equation 6

$$\delta_1^i = \mu_1^i - \hat{\mu}_1^i$$

and

Equation 7

$$\sigma_2 = \frac{\hat{\pi}_1^i}{\hat{\pi}_2^i \hat{\pi}_1^i + 1}$$

Where π is the precision (i.e., the inverse variance) in participants' posterior belief $\frac{1}{\sigma}$, and δ_1^i is the prediction error on the trial outcome. Caret symbols (^) are used to denote predictions *prior* to observing the outcome at trial *i*. Thus, $\hat{\pi}_1^i$is the precision of the prediction at the first hierarchical level and $\hat{\pi}_2^i$ is the precision of the prediction of the posterior belief. It can be shown from **Equation 7** that prediction errors are given a larger weight when the precision of the prediction of the agent's choice is high, or when the precision of the belief about the agent's preference (i.e., exchange rate between money and pain) is low. In summary, these equations describe trial-wise updating of beliefs about an agent's preference towards harming the victim, which approximates Bayes optimality (in an individualized sense given differences in ω) and determines the participant's estimate of the probability that an agent will harm. Crucially, our model provides a trial-by-trial estimate of the subject's uncertainty about the agent's preference towards harming the victim as measured by the variance of beliefs, σ. The variance weights predictions errors on a trial-by-trial basis and thus represents a *dynamic* learning rate because it accounts for the precision of the belief at any given time.

Decision model. The decision model describes how the participant's posterior belief about the agent's preference maps onto their predictions of the agent's decisions (*y*). In the HGF, this belief $\hat{\mu}_1^i$corresponds to the logistic sigmoid transformation of the predicted preference $\mu_2^{i-1}$of the agent towards harming the victim.

Equation 8

$$\hat{\mu}_1^i = s(\mu_2^{i-1})$$

For the present study, we assumed that participants would predict others' decisions using a similar rationale to how they make decisions themselves. In other words, we assumed that people's preferences are described by a utility model, and that people think others' preferences are described by the same model. Consequently, we applied a decision model that accurately describes human choices in the same choice setting (4–6).

Equation 9

$$V_{\text{harm}}^i = \left(1 - \hat{\mu}_1^i\right) \Delta \mathrm{m}^i - \hat{\mu}_1^i \Delta \mathrm{s}^i$$

This applied the predicted belief about the agent's preference derived from the perceptual model $\hat{\mu}_1^i$ to compute the value that the agent will choose the more harmful option on trial *i,* given the difference in money (Δm) and shocks *(*Δs) between the two options. The probability that the participant predicts the more harmful option ($y = 1$) as opposed to the more helpful option ($y = 0$) is described by the softmax function in **Equation 10**.

Equation 10

$$P_{\text{harm}}^i = s(\beta V_{\text{harm}}^i)$$

Where β is a free parameter (individually estimated like ω) that describes how sensitive predictions are to the relative utility of different outcomes, or the prediction noise.

Estimation of model parameters. A crucial aspect of Bayesian inference is the specification of a prior distribution for the belief (listed in **Supplementary Table** S**1**). We defined the priors based on previous research using the same experimental design. Specifically, in keeping with our experimental design, which did not give participants any basis for assumptions about the agent's tendency to harm, we chose to initialize the prior mean over μ_2 and σ_2. such that it amounted to a neutral prior belief about κ which was equidistant from the true value of the agents' preferences. For the free parameters ω and β, we chose a prior mean that was relatively uninformative (with large variance) to allow for substantial individual differences in learning both between participants and within participants (i.e. between agents).

Supplementary Table S1

Prior mean and variance of the perceptual and response model parameters.

Parameter	Notes		mean	variance
**ω**	Constant component of the tonic volatility at the second level. Represents the temporal evolution of x_2. *Estimated in native space.*		-4	1
**Predictions (x_1)**	Predictions are a sigmoid transformation of x_2 , and so do not have prior values.	μ_1:	none	none
		σ_1:	none	none
**Probabilities (x_2)**	The prior mean on x_2 (prior belief about agent's harm-aversion, κ) was fixed to a neutral point that was equidistant from the true κ value of both agents. Estimated in logit space.	μ_2:	0.5	0
	The prior variance on x_2 was fixed to ensure that any differences in learning about good and bad agents derived from the model could not result from differences in the prior estimates. Estimated in log-space.	σ_2:	0.35	0
**β**	Constant component that describes how sensitive prior beliefs are to the relative utility of different outcomes, or the prediction noise. Estimated in log-space.		1	1

The perceptual model parameter ω and decision model parameter β were estimated from the trial-wise predictions using the Broyden Fletcher Goldfarb Shanno optimization algorithm as implemented in the HGF Toolbox (https://tnu.ethz.ch/tapas). This allowed us to obtain the maximum-a-posteriori estimates of the model parameters and provided us with state trajectories and parameters representing an ideal Bayesian observer given the individually estimated parameter ω.

We fit the model separately for participant's predictions of the bad and good agent. This produced for each agent a sequence of trial-wise beliefs about the agent's preference ($\hat{\mu}_1^i$), as well as the precision of each belief (σ^i), and two participant-specific parameters, ω and β. to the temporal emphasis of belief stability in BPD, we focus out analysis on variance of beliefs σ, which reflects a dynamic learning rate dictating trial-by-trial belief updating as a function of the precision (i.e., inverse uncertainty) of beliefs about the agent's moral preference.

Additional Measures

Borderline evaluation of severity over time (BEST). We used the BEST (7) to assess the severity of BPD symptomology in participants with BPD at the time of participation. The BEST is a 15-item questionnaire which measures thoughts, emotions, and behaviors (positive and negative) typical of BPD. Positive behaviors were not measured in this study, and thus participants responded to only 12 of the 15 items. Each item asks participants to rate their experience with each of the items since their last clinical session; the lowest score of 1 means that it caused little or no

problems, and the highest score of 5 means that it caused extreme distress, severe difficulties with relationships, and/or kept them from completing tasks. The scores from the 12 items were added together to yield a score between 12 and 60, where higher scores indicated greater BPD severity.

Personality Inventory for DSM-5, brief form (PID-5-BF). We used the PID-5-BF (8), a 25-item self-report questionnaire, to assess clinically relevant personality traits that do not necessarily constitute a personality disorder. The PID-5-BF constitutes five personality trait domains: negative affect, detachment, antagonism, disinhibition, and psychoticism. Each item on the questionnaire asks participants to rate how well the item describes him or her generally on a scale from 0 (*very false or often false*) to 3 (*very true or often true*). The scores from all items were added together to produce a score between 0 and 75, with higher scores indicating greater general overall personality dysfunction.

McLean Screening Instrument for BPD (MSI). The MSI (9) was used as a screening measure for the presence of clinically relevant BPD in the control group. The validated instrument consists of ten true-false self-report questions to assess the occurrence of symptoms typically found in BPD, such as "*Have you deliberately hurt yourself physically (e.g. punched yourself, cut yourself, burned yourself)*". The screen is regarded as positive when seven or more of the symptoms are true.

Self Report Psychopathy - Revised, short form (SRP-R-SF). We used the SRP-R-SF (10), a 29-item self-report questionnaire, to assess psychopathic personality traits across BPD participants and non-BPD control participants. The instrument constitutes four factors of psychopathy: affective callousness, interpersonal manipulation, antisociality, and erratic lifestyle. Each item on the questionnaire asks participants to rate the extent to which they thought the item reflected their own beliefs using a 5-point likert scale (1 = *strongly disagree* to 5 = *strongly agree*). The scores from all items were added together to produce a total psychopathy score, with higher scores indicating greater general overall psychopathic personality traits.

Structured Clinical Interview for axis II disorders (SCID-II). The SCID-II is a semi-structured clinical interview administered by trained clinical and designed to asses a clinical diagnosis of axis II disorders consistent with the DSM-IV. The SCID-II was used to establish a clinical diagnosis of BPD in untreated BPD and DTC-treated participants.

Supplemental Results

Motivation to accurately predict the agents' choices. Because non-BPD and BPD participants completed the task under very different experimental settings (non-BPD participants: conducted online, BPD participants: conducted in the clinic), we wanted to verify that the groups were equally motivated to learn about the agents and predict their decisions. Consequently, after predicting all the choices for a given agent, we explicitly asked participants to indicate on a continuous scale from 0 (*very unmotivated*) to 100 (*very motivated*) "How motivated to be accurate did you feel during the task?". We additionally calculated the percent of choices accurately predicted by each participant and compared between groups. We confirmed that BPD and non-BPD participants were similarly accurate (% accuracy: bad: Z = -1.103, p = 0.270; good: Z = 0.295, p = 0.768) and motivated in their predictions (motivation rating: bad: Z = - 0.879, p = 0. 379; good: Z = -1.704, p = 0.088).

Model validation. Three computational models were compared to describe how participants learned the agents' preferences and predicted their choices. We fit the HGF (2,3), which identified participant-specific parameters to describe each individual participant's learning process. Beliefs about an agent's harm preference were updated using a Bayesian reinforcement learning algorithm, with precision-weighted prediction errors driving belief updating at the different levels of the hierarchical model. Second, we fit a Rescorla Wagner model, in which beliefs were updated by prediction errors with a fixed learning rate. Third, we fit a modified Rescorla Wagner model, in which beliefs were updated by prediction errors with separate fixed learning rates for helpful and harmful outcomes. For details about the alternative models, see **Supplementary Table 2**.

Supplementary Table S2

Details of alternative models for model comparison

Model	Notes	Estimated parameters
Rescorla Wagner with one learning rate	Beliefs are symmetrically updated, with a single learning rate for each participant.	α = Learning rate<br>β = Prediction noise
Rescorla Wagner with two learning rates	Beliefs are asymmetrically updated, with separate learning rates for positive versus negative outcomes, for each participant.	α_{pos} = Learning rate positive outcomes<br>α_{pos} = Learning rate negative outcomes<br>β = Prediction noise
HGF	A two level model, with one estimated parameter governing the volatility of beliefs at the second level, and a second estimated parameter governing the prediction noise.	ω = Tonic volatility<br>β = Prediction noise

The log-model evidence (LME) indicated that the HGF model (sum LME = -7149) outperforms both a simple single learning rate RW model (sum LME = -7444) and a RW model with separate learning rates for positive and negative outcomes (sum LME = -7192). We validated these findings using formal Bayesian Model Selection. To this end, we used LME data to compare between the HGF and our two RW models. This analysis yielded a protected exceedance probability indistinguishable from 1 for the HGF model for both agents, indicating effectively a 100% probability that the HGF model better explains the data than the other models included in the comparison.

Subjective uncertainty ratings in BPD versus non-BPD and DTC-treated participants. For completeness, we performed an omnibus robust linear regression analysis on subjective uncertainty ratings that included all three groups (BPD, non-BPD, and DTC) in a single model, where group was dummy coded with BPD as the reference group. Tests of group effects were conducted using Bonferroni adjusted alpha levels of .025 to account for multiple comparisons. The analysis yielded a significant main effect of agent (β = -0.155±0.073, t = 2.126, p = .034), indicating that participants held more uncertain impressions of the bad agent than the good agent. Overall, BPD participants uncertainty ratings did not significantly differ from non-BPD participants (β = -0.098±0.056, t = -1.739, p = .082), or DTC-treated participants (β = -0.089±0.071, t = -1.258, p = .209). The effect of agent was significantly smaller in BPD participants, relative to both non-BPD participants (β = 0.264±0.080, t = 3.310, p < .001) and DTC-treated participants (β = 0.266 ±0.100, t = 2.665, p = .008), as indicated by significant interactions between agent and group.

Learning rates in BPD versus non-BPD and DTC-Treated participants. We performed an omnibus robust linear regression analysis on learning rates that included all three groups (BPD, non-BPD, and DTC) in a single model, where group was dummy coded with BPD as the reference group. Again, this analysis yielded a significant main effect of agent (β = -0.831±0.023, t = 36.888, p < .001), indicating that participants updated beliefs about the bad agent at a faster rate than the good agent. Overall, learning rates for the untreated BPD participants did not differ from non-BPD participants (β = -0.001±0.017, t = -0.060, p = .953), or DTC participants (β = -0.024±0.022, t = -1.103, p = .270). However, relative to untreated BPD participants, the effect of agent on learning rates was significantly larger relative to both non-BPD participants (β = 0.113±0.025, t = 4.607, p < .001) and DTC-treated participants (β = 0.319 ±0.031, t = 10.355, p < .001), as indicated by significant interactions between agent and group.

Subjective moral impressions in BPD versus non-BPD participants. Examining subjective impression ratings revealed that participants formed more negative impressions about the 'bad' agent than the 'good' agent (mean±SEM, β = -1.178 ± 0.027, t = -44.299, p < .001). The main effect of group (β = -0.041 ± 0.047, t = -.872, p = .383) and the interaction between agent and group were not significant (β = -0.441 ± 0.067, t = -1.706, p = .088). Thus, the valence of moral impressions did not vary as a function of BPD diagnosis.

Subjective uncertainty ratings in BPD versus DTC-treated participants. Examining subjective uncertainty ratings yielded a significant main effect of agent (β = 0.156±0.070, t = 2.240, p = .025), indicating that participants held more uncertain impressions of the bad agent than the good agent. DTC-treated and untreated BPD participants were similarly uncertain about their impressions overall (β = -0.085 ± 0.067, t = -1.265, p = .206). However, we found that DTC-treated BPD participants, relative to untreated BPD participants, showed more uncertain impressions of the bad agent (β = 0.188±0.067, t = 2.802, p = .005; **Figure 3a**) as indicated by significant interactions between agent and group (β = 0.277±0.095, t = 2.904, p = .003).

Learning rates in BPD versus DTC-Treated participants. Again, we observed a significant main effect of agent on learning rates (β = 0.153±0.037, t = 4.115, p < .001), indicating that BPD participants updated beliefs about the bad agent at a faster rate than the good agent. Overall, learning rates for the DTC-treated and untreated BPD participants did not significantly differ (β = -0.031 ± 0.036, t = -0.870, p = .384). However, we found that DTC-treated BPD participants, relative to untreated BPD participants, showed faster learning rates for the bad agent (β = 0.543±0.040, t = 13.698, p < .001; **Figure 3b**), as indicated by significant interactions between agent and group (β = 0.589±0.052, t = 11.588, p < .001).

Effect of individual differences in the severity of BPD symptomology on subjective uncertainty ratings and learning rates. We used a robust linear regression model that included the effects of agent (bad, good), and Borderline Evaluation of Severity over Time (BEST) scores, and their interaction (controlling for trial number) to investigate their effects on subjective uncertainty ratings and learning rates. Consistent with prior findings, participants overall held more uncertain impressions of the bad agent than the good agent (main effect of agent: β = 0.904±0.171, t = 5.272, p < .001) and faster learning rates for the bad agent than the good agent (β = 1.308±0.052, t = 25.193, p < .001). However this effect decreased with increasing BPD symptomology (interaction between agent and BEST: *uncertainty rating,* β = -0.018±0.005, t = -3.784, p < .001; *learning rate*, β = -0.004±0.001, t = -2.821, p = .005). Specifically, higher BEST scores were associated with less uncertain impressions of the bad agent (β = -0.012 ± 0.003, t=-3.262, p = .001), though the effect on learning rates did not reach significance (β = -0.003 ± 0.002, t=-1.514, p = .130). Higher BEST scores were associated with *more* uncertain impressions of the good agent (β = 0.007 ± 0.003, t=2.078, p = .038), and faster belief updating (β = 0.003 ± 0.001, t=6.118, p < .001).

Prior expectations in moral inference. BPD participants expressed more pessimistic expectations about the agents' moral behavior than non-BPD participants. Thus, a plausible explanation for more certain beliefs about bad agents and less certain beliefs about good agents is that the good agent violated BPD participants' expectations to a greater degree than the bad agent. In other words, the bad agent's behavior would be more consistent with patient's prior expectation (and therefore increase confidence and rigidity of posterior beliefs) while the good agent's behavior would be less consistent with patient's prior expectations (thus, decrease confidence and rigidity of posterior beliefs).

Previous work suggests that prior moral expectations are unlikely to impact the ability to adapt learning as a function of moral information in healthy adults (1). Human may have evolved to adapt learning according to moral information to aid survival. In turn, this adaptive mechanism may enable healthy adults to discount expectations to build richer models of agents when harmful outcomes are expected (i.e., in response to negative moral expectations). One possibility is that patients with BPD lack the mechanism for adapting learning according to moral information. That is, while healthy adults may be able to override prior expectations and rapidly adjust their learning for putatively bad agents, this adaptive mechanism may be absent in BPD. As a result, learning may be more sensitive to prior expectations in BPD. If this is the case, we would expect learning in BPD to be more strongly influenced by prior moral expectations than learning in non-BPD participants.

In line with this prediction, we found a significant three-way interaction between prior expectations, BPD diagnosis, and agent (β = 0.004±0.002, t = 2.214, p = .027). To unpack the interaction, we performed a similar regression splitting the data as a function of BPD diagnosis. Consistent with previous findings (1), prior expectations were not associated with differences in learning rates between the good and bad agent in non-BPD control participants (β = -0.001±0.001, t = -1.454, p = .146; **Supplementary Figure S1**). Conversely, prior expectations predicted asymmetric learning rates for good and bad agents in BPD participants: more pessimistic expectations were associated with a smaller learning asymmetry (β = 0.003±0.001, t = 2.250, p = .025; **Supplementary Figure S1**). The findings provide preliminary evidence to suggest that the mechanisms underlying the ability to rapidly adapt learning towards moral information in healthy adults may be absent in BPD.

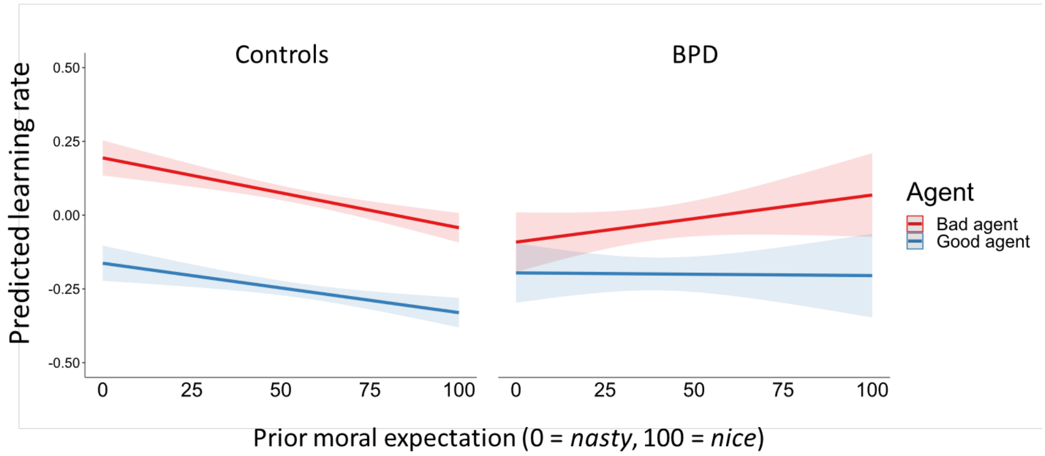

Supplementary Figure S1. Prior moral expectations moderate belief updating in BPD. Effect of prior moral expectations on estimated learning rates for the control (i.e., non-BPD) group (left) and BPD group (right). Prior moral expectations were measured on a continuous scale before

observing any of the agent's choices. The scale asked participants to indicate how nasty or nice they expected the agents would be in the task. Error bands represent 95% confidence intervals.

Prior expectations did not significantly differ between DTC-treated and untreated BPD participants. Nonetheless, we performed a similar regression analysis to explore the three-way interaction and observed a significant interaction between prior expectations, agent, and group on learning rates (β = -0.010±0.002, t = -4.752, p < .001; **Supplementary Figure S2**). To unpack the interaction, we fit the regression model separately for untreated BPD and DTC treated participants. Again, we found that worse expectations were associated with smaller asymmetric updating between agents in the BPD group (β = 0.003±0.001, t = 2.250, p = .025). However, the opposite pattern was observed for the DTC treated group: worse expectations were associated with larger asymmetric updating between agents (β = -0.007±0.002, t = -4.615, p < .001). These findings suggest that even though DTC-treated and untreated BPD groups had similar moral expectations, the groups differed in how expectations subsequently shaped learning.

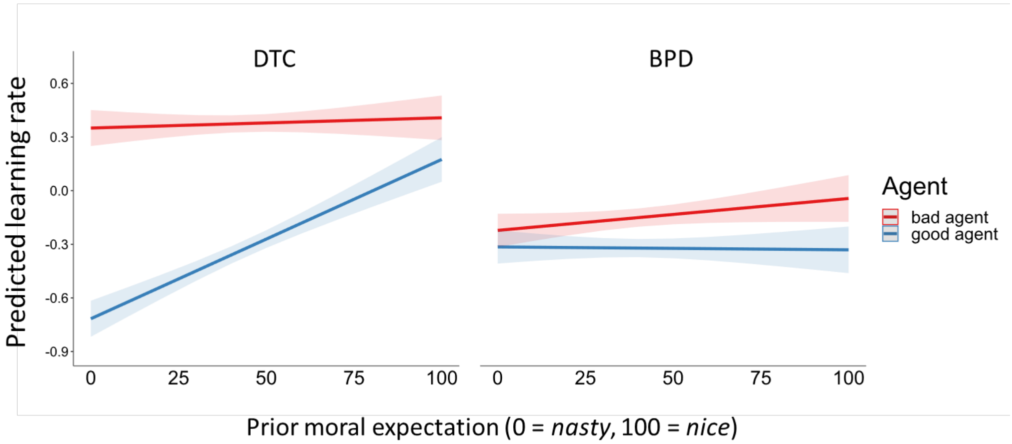

Supplementary Figure S2. Prior moral expectations moderate belief updating. Effect of prior moral expectations on estimated learning rates for the DTC group (left) and BPD group (right). Prior moral expectations were measured on a continuous scale before observing any of the agent's choices. The scale asked participants to indicate how nasty or nice they expected the agents would be in the task. Error bands represent 95% confidence intervals.

BPD, medication use, and moral inference. A supplementary analysis investigated the interaction between group (DTC vs. BPD) and agent (bad vs. good) on subjective uncertainty and learning rates, controlling for medication use. Medication use was entered into the regression as a dummy variable and indicated whether the participants were receiving psychotropic or antidepressant medication during the time of participation. Medication use did not significantly predict subjective uncertainty ratings (β = 0.027±0.048, t = 0.551, p = .582) nor did patient group (β = -0.082±0.068, t = -1.201, p = .230). Overall, participants were more uncertain about their impressions of the bad agent relative to the good agent (β = 0.156±0.070, t = 2.240, p = .025). The

interaction between group and agent on subjective uncertainty remained significant after controlling for medication use (uncertainty: β = 0.277±0.095, t = 2.898, p = .004; learning rates: β = 0.577±0.050, t = 11.441, p < .001). Relative to untreated BPD participants, DTC-treated participants were more uncertain about their impressions of the bad agent (β = 0.183±0.068, t = 2.691, p = .007) but did not significantly differ in their uncertainty about their impressions of the good agent (β = -0.068±0.069, t = -0.998, p = .318).

Patient group did not significantly predict overall learning rates (β = -0.051±0.036, t = -1.427, p = .154). Medication use was associated with slower learning rates overall (β = -0.180±0.026, t = -7.050, p < .001) and participants had higher learning rates for the bad agent relative to the good agent (β = 0.169±0.037, t = 4.587, p < .001). Notably, the interaction between group and agent on learning rates remained significantly after controlling for medication use (β = 0.577±0.050, t = 11.441, p < .001). Relative to untreated BPD participants, DTC-treated participants had higher learning rates for bad agent (β = 0.516±0.040, t = 12.853, p < .001) but marginally lower learning rates for the good agent (β = -0.058±0.030, t = -1.922, p = .055).

Supplemental References

1. Siegel JZ, Mathys C, Rutledge RB, Crockett MJ (2018): Beliefs about bad people are volatile. *Nature Human Behaviour* 2: 750.

2. Mathys C, Daunizeau J, Friston KJ, Stephan KE (2011): A Bayesian foundation for individual learning under uncertainty. *Front Hum Neurosci* 5: 39.

3. Mathys C, Lomakina E, Daunizeau J, Iglesias S, Brodersen K, Friston K, Stephan KE (2014): Uncertainty in perception and the Hierarchical Gaussian Filter. *Front Hum Neurosci* 8: 825.

4. Crockett MJ, Siegel JZ, Kurth-Nelson Z, Ousdal OT, Story G, Frieband C, *et al.* (2015): Dissociable Effects of Serotonin and Dopamine on the Valuation of Harm in Moral Decision Making. *Curr Biol* 25: 1852–1859.

5. Crockett MJ, Kurth-Nelson Z, Siegel JZ, Dayan P, Dolan RJ (2014): Harm to others outweighs harm to self in moral decision making. *PNAS* 111: 17320–17325.

6. Crockett MJ, Siegel JZ, Kurth-Nelson Z, Dayan P, Dolan RJ (2017): Moral transgressions corrupt neural representations of value. *Nat Neurosci* 20: 879–885.

7. Pfohl B, Blum N, St. John D, McCormick B, Allen J, Black DW (2009): Reliability and validity of the borderline evaluation of severity over time (BEST): a self-rated scale to measure severity and change in persons with borderline personality disorder. *J Pers Disord* 23: 281–293.

8. Krueger RF, Derringer J, Markon KE, Watson D, Skodol AE (2012): Initial Construction of a Maladaptive Personality Trait Model and Inventory for DSM-5. *Psychol Med* 42: 1879–1890.

9. Zanarini MC, Vujanovic AA, Parachini EA, Boulanger JL, Frankenburg FR, Hennen J (2003): A screening measure for BPD: the McLean Screening Instrument for Borderline Personality Disorder (MSI-BPD). *J Pers Disord* 17: 568–573.

10. Neumann C, Pardini D (2012): Factor Structure and Construct Validity of the Self-Report Psychopathy (SRP) Scale and the Youth Psychopathic Traits Inventory (YPI) in Young Men. *Journal of Personality Disorders* 28: 419–433.
